# Internet Addiction, *Hikikomori* Syndrome, and the Prodromal Phase of Psychosis

**DOI:** 10.3389/fpsyt.2016.00006

**Published:** 2016-03-03

**Authors:** Emmanuel Stip, Alexis Thibault, Alexis Beauchamp-Chatel, Steve Kisely

**Affiliations:** ^1^Département de Psychiatrie, Faculté de Médecine, Université de Montréal, Montréal, QC, Canada; ^2^Centre Hospitalier de l’Université de Montréal, Hôpital Notre-Dame, Montréal, QC, Canada; ^3^School of Medicine, University of Queensland, Brisbane, QLD, Australia; ^4^Department of Psychiatry, Dalhousie University, Halifax, NS, Canada; ^5^Department of Community Health and Epidemiology, Dalhousie University, Halifax, NS, Canada

**Keywords:** hikikomori, Internet addiction, schizophrenia, social withdrawal, prodromal phase

## Abstract

Computers, video games, and technological devices are part of young people’s everyday lives. Hikikomori is a Japanese word describing a condition that mainly affects adolescents or young adults who live isolated from the world, cloistered within their parents’ homes, locked in their bedrooms for days, months, or even years on end, and refusing to communicate even with their family. These patients use the Internet profusely, and only venture out to deal with their most imperative bodily needs. Although first described in Japan, cases have been described from around the world. This is the first published report from Canada. The disorder shares characteristics with prodromal psychosis, negative symptoms of schizophrenia, or Internet addiction, which are common differential or comorbid diagnoses. However, certain cases are not accompanied by a mental disorder. Psychotherapy is the treatment of choice although many cases are reluctant to present. The exact place of hikikomori in psychiatric nosology has yet to be determined. We searched Medline up to 12th May, 2015 supplemented by a hand search of the bibliographies of all retrieved articles. We used the following search terms: Hikikomori OR (prolonged AND social AND withdrawal). We found 97 potential papers. Of these 42 were in Japanese, and 1 in Korean. However, many of these were cited by subsequent English language papers that were included in the review. Following scrutiny of the titles and abstracts, 29 were judged to be relevant. Further research is needed to distinguish between primary and secondary hikikomori and establish whether this is a new diagnostic entity, or particular cultural or societal manifestations of established diagnoses.

## Introduction

Adolescence is a time of transition and the age of onset of many psychiatric disorders. Typically, early symptoms are insidious and non-specific, such as social withdrawal and isolation. At a time where new technologies disrupt people’s life and usual means of interactions with others, it may be difficult to distinguish between what is developmentally normal and what represents the start of a wide range of disorders, including depression, social phobia, personality disorders, schizophrenia, Internet addiction, or *hikikomori*. Since 1970s, Japan has seen the emergence of a particular type of severe social withdrawal termed *hikikomori*, a Japanese word describing psychosocial and familial pathology (1, 2). *Hikikomori* comes from the verb *hiki*, which means to move back, and *komoru*, which means to come into (3). The disorder mainly affects adolescents or young adults who live cut off from the world, cloistered within their parents’ homes, locked in their bedrooms for days, months, or even years on end. They refuse to communicate even with their family, use the Internet profusely, and only venture out to deal with their most imperative bodily needs. Many *hikikomori* turn to the Internet, and sometimes spend more than 12 h a day in front of the computer. As a consequence, more than half of patients are at-risk of Internet addiction, and approximately one-tenth would fit diagnostic criteria for such an addiction (4).

The concept of *hikikomori* is controversial. A major issue is the absence of a clear definition and no consensus on diagnostic criteria across studies (5). There is debate as to whether this syndrome marks a culture-specific response to societal change in Japan (6) or whether it is an emerging psychiatric disorder that may be present elsewhere (7). It is even suggested that *hikikomori* could be beneficial to these individuals in whom it can help regain a sense of identity and social connectedness through new means more suited to them (6). Another area of controversy is whether *hikikomori* should be diagnosed if another psychiatric disorder can account for the symptoms. Some authors argue that the term “secondary *hikikomori*” should be used if comorbidity is present and at least partially explains the syndrome, while in the absence of an intercurrent psychiatric diagnosis, the term “primary hikikomori” should be used (5).

Although first described in Japan, cases have been described from around the world. This is the first published report from Canada.

## Case Description

This is the case of a young man living in Montréal aged 21 years, Caucasian, without medical antecedents other than sleep rituals in the form of rhythmic movement disorder (rocking) for which he had successfully sought behavioral treatment at age 13. His physical workup was normal. He smoked one pack of cigarettes a day and took no other drugs. He was studying engineering at university; he had always been a bright student. He played sports.

The problems started when he lost an academic competition 1 year, after always being accustomed to succeed in his studies. Although he did not feel depressed, the young man spent more and more time alone in his room. He no longer joined his family for meals as usual, preferring to grab something from the refrigerator and return to his room immediately, where he spent most of the day at the computer. The first year, he stayed in a fairly spacious, well-equipped bedroom, eating meals that were prepared for him but declining to join the family at table. However, he subsequently left the family home to live alone in a small apartment. There, he ended up almost cutting off contact with his family except for doing laundry and getting a check or a meal from time to time. However, he did wash regularly.

He spent his time on the Internet or playing video games in complete social isolation, although he claimed that he was still going to his university classes. The situation worried his family and friends, who tried confiscating his computer for several weeks, because he was spending more than 12 h a day in front of the computer, essentially to play games or watch video clips. This confiscation had no effect on his isolation and social withdrawal. His family asked him to go for counseling, but he refused to do so, and only the family members sought help. The patient did not feel sad or suicidal and refused to seek help.

Then, he experienced another failure at university. It was decided, with the young man’s agreement – indeed, almost at his request, given his sense of failure – that he should again reside with a member of his family. His behavior briefly improved, but by the second year, he again began to spend more than 15 h a day at the computer. He stopped attending class although he realized that this would lead to failure. He became aggressive and irritable more often when his family tried to discuss his behavior and again refused requests that he seek treatment. All of this ended in a complete break with his family, whereupon they adopted more authoritarian measures.

After dropping out of school and deadlock concerning finances, the young man became more open to change. His mental examination could almost be defined as normal, other than some obsessive-compulsive traits, signs of emotional numbing and social withdrawal, and elements of social phobia and anxiety about new things. There was no evidence of depression, suicidal ideation, psycho-sensory phenomena, or delirium. His cognition was normal, and he had partial insight into the possible reasons for his withdrawal. He justified it as a way of being free and referred to intergenerational misunderstanding. The results of his neurological examination were normal, including an MRI. With supervision, he resumed his work and studies without the need for medication or formal psychotherapy.

## Literature Review

We searched Medline up to 12th May, 2015 supplemented by a hand search of the bibliographies of all retrieved articles. We used the following search terms: Hikikomori OR (prolonged AND social AND withdrawal). We found 97 potential papers. Of these 42 were in Japanese, and 1 in Korean. However, many of these were cited by subsequent English language papers that were included in the review. Following scrutiny of the titles and abstracts, 29 were judged to be relevant. We were unable to obtain six of these papers. We also found a relevant book in French (8).

### Prevalence

*Hikikomori* has been defined by a Japanese expert group as having the following characteristics: (1) spending most of the time at home; (2) no interest in going to school or working; (3) persistence of withdrawal for more than 6 months; (4) exclusion of schizophrenia, mental retardation, and bipolar disorder; and (5) exclusion of those who maintain personal relationships (e.g., friendships) (9, 10). Other criteria are more controversial. These include the inclusion or exclusion of psychiatric comorbidity (primary versus secondary *hikikomori*), duration of social withdrawal, and the presence or absence of subjective distress and functional impairment (5).

Approximately 1–2% of adolescents and young adults are *hikikomori* in Asian countries, such as Japan, Hong Kong, and Korea (4, 9, 11). Most cases are males (8–13) with a mean duration of social reclusion ranging from 1 to 4 years, depending on study design and setting (5, 8, 13, 14). Comorbidity with other psychiatric diagnosis is also very variable, ranging from none (13), half of cases (11), to almost all cases (12, 13). This variability may be explained by the lack of consensus on the definition of *hikikomori* and also because different recruitment methods were used across studies. However, there seems to be an emerging consensus that a majority of *hikikomori* cases have comorbid psychiatric diagnoses (5).

*Hikikomori* was originally described in Japan, but cases have subsequently been reported in Oman (15), Spain (13, 16, 17), Italy (18), South Korea (4, 14), Hong Kong (19), India (20), France (8, 21), and the United States (19, 22). Aside from case reports, surveys of psychiatrists from countries as diverse as Australia, Bangladesh, Iran, Taiwan, and Thailand suggest *hikikomori* cases are seen in all the countries examined, especially in urban areas (23).

There are few well-designed observational studies of *hikikomori*. Most of what is known is derived from small studies with non-representative sample. More importantly, there is little information on the prevalence or characteristics of *hikikomori* outside a few countries in Asia.

Aside from the lack of clear definition of the syndrome, the resulting social isolation (11) and the shame and guilt of the family, are all barriers to the identification and characterization of these individuals. Of note, the same factors also cause long delays in receiving treatment (1, 4, 5, 10, 13).

### Etiology of *Hikikomori* and Links to Internet Use

Consensus on the etiology of *hikikomori* has not been reached, and there are several possible explanations. On a psychological level, numerous reports and articles mention the association between *hikikomori* and aversive, even traumatic, childhood experiences. It seems many of the cases experienced social exclusion as children, often having been victims of bullying at school or other forms of peer rejection (4–6, 8, 10, 12, 15, 24, 25). An introverted personality, temperamental shyness, and an ambivalent or avoidant attachment style may also predispose to develop *hikikomori* (5, 20, 25).

At a familial and environmental level, there may be a link between the emergence of the disorder and dysfunctional family dynamics (4, 8, 10, 19, 26), parental rejection (25) or overprotection (5), and parental psychopathology (13, 27). Poor academic achievement, combined with high expectations, and sometimes subsequent school refusal, also seem to be factors in the development of *hikikomori* (3–6).

Sociocultural explanations, including a breakdown of social cohesion, urbanization, technological progress, globalization, and downward social mobility, may also have a role in the emergence of *hikikomori* (5, 8, 11, 16, 28, 29). These changes may lead to disengagement or dissociation from society in predisposed individuals as a psychic response to painful emotions. As such the condition forms one part of a spectrum of social dissociative problems ranging from disengagement from conventional social roles (*makeinu*) to school refusal (*futoko*) and ultimately complete social withdrawal (*hikikomori*).

The invention of the Internet and subsequent changes to the way people interact with and within society may also be major factors contributing to *hikikomori* (26). For instance, a preference for online communication may play a role in the development of social withdrawal in certain individuals (26).

### Differential Diagnosis of *Hikikomori*

Differentiating between *hikikomori* and the early stage of other psychiatric disorders can be difficult since many of the symptoms are non-specific and can be found across various conditions (21, 30). These include isolation, social deterioration, loss of drive, dysphoric mood, sleep disorders, and reduced concentration (21, 30, 31). As mentioned earlier, although comordibity with psychiatric diagnosis vary depending on study methodology and sampling, the few observational studies and recent reports in the literature seem to agree on a high proportion of such diagnoses. These are most commonly schizophrenia, other psychotic disorders, and mood or anxiety disorders, such as major depression and social phobia (2, 8, 9, 12, 13, 32). Others have suggested autism spectrum disorder, personality disorders, such as schizoid or avoidant disorders, or cannabis abuse with amotivational syndrome, or even Internet addiction (5, 8–10, 23). In the following sections, *hikikomori will be compared to Internet addiction and psychosis*.

#### Comparison between Hikikomori and Internet Addiction

Like *hikikomori*, Internet addiction is an emerging psychiatric diagnosis, and the definition and clinical features are still a matter of debate. Table 1 presents proposed diagnostic criteria that have been validated in a large sample of Chinese participants (*n* = 405) (34).

**Table 1:** **Internet addiction diagnostic criteria (33)**.

**A. Symptom criterion**
All the following must be present
1. Withdrawal, as manifested by a dysphoric mood, anxiety, irritability, and boredom after several days without Internet activity
2. Preoccupation with the Internet (thinks about previous online activity or anticipates next online session)
At least one (or more) of the following
3. Tolerance, marked increase in Internet use required to achieve satisfaction
4. Persistent desire and/or unsuccessful attempts to control, cut back, or discontinue Internet use
5. Continued excessive use of Internet despite knowledge of having a persistent or recurrent physical or psychological problem likely to have been caused or exacerbated by Internet use
6. Loss of interests, previous hobbies, entertainment as a direct result of, and with the exception of, Internet use
7. Uses the Internet to escape or relieve a dysphoric mood (e.g., feelings of helplessness, guilt, and anxiety)
**B. Exclusion criterion**
Excessive Internet use is not better accounted for by psychotic disorders or bipolar I disorder
**C. Clinically significant impairment criterion**
Functional impairments (reduced social, academic, and working ability), including loss of a significant relationship, job, educational, or career opportunities
**D. Course criterion**
Duration of Internet addiction must have lasted for an excess of 3 months, with at least 6 h of Internet usage (non-business/non-academic) per day

These criteria are still tentative since no major nosographical system has adopted them so far. DSM-5 has introduced a similar diagnosis, termed Internet gaming disorder, as a condition requiring further study. Gaming disorder shares the first six of the above criteria, but adds four further criteria: continued use despite the patient knowing it is problematic, lying to the family about use, use of Internet to escape negative mood, and social/interpersonal/vocational problems due to the disorder (35). Other differences are that there are no exclusion criterion in the DSM classification, the duration is 12 months instead of 3 months, patients need to meet five criteria to receive the diagnosis and, even more importantly, the diagnosis is limited to Internet gaming and does not take into account other Internet activities.

The epidemiology of Internet addiction is unclear because criteria are still debated, population-based epidemiological studies are rare, and Internet use has increased tremendously since it was first described. Tao et al. (33) reported a prevalence ranging from 1 to 14%, citing studies done in 2008 and 2009. Since then, social media use (*Instagram*, *Facebook*, etc.) and *YouTube* has become widespread and could have lead to further increase in problematic Internet use. Shek et al. (36) found a prevalence of 17–26.8% in adolescents in Hong Kong. This is far more than *hikikomori* that is estimated to affect 1–2% of the population in Asia (see above). It is difficult to know what is the age of onset since most studies have been done with adolescents or young adults and children are now exposed to Internet from a very young age. Problematic use could start before adolescence. That is, in sharp contrast with *hikikomori* that tend to occur later in adolescence of young adulthood [average age of onset of 22.3 years in Ref. (9)]. A national survey in Korea found that adolescent boys were more likely to be addicted than girl (3.6 versus 1.9%) (37), which is consistent with *hikikomori*. In both cases, Asian countries seem to be at the forefront of research.

The choice of the term “addiction” highlights a presumed link between Internet problematic use and other behavioral addiction (such as gambling) and substance addiction. Internet-addicted individuals would be three time more likely than non-addicted to suffer from alcohol abuse (38). Brand and Laier (39) reviewed existing neuroimagery studies on Internet addiction and found a similar pattern of nucleus accumbens/orbitofrontal cortex overstimulation than in substance-addicted individuals. Common etiological models of Internet addiction are thus inspired by this presumed resemblance. In Ref. (40), four main models were extracted from the literature: the learning theory model (positive and negative reinforcers), the cognitive-behavioral model, the social skills deficit model, and the reward-deficiency hypothesis (Internet would deliver stronger stimuli than real life, attracting people who need more intense stimuli). Intrapersonal factors (e.g., self-esteem, emotional difficulties, impulse control, etc.) are greater risk factors than interpersonal ones (e.g., social anxiety, problematic peer relations, parent relationship difficulties, family functioning, etc.) according to a large and recent meta-analysis (41). It has been suggested that both conditions represent a dissociative response to painful emotional states (33, 42). While reinforcement could play a role in hikikomori too, interpersonal factors have been more consistently reported in hikikomori, which contrast with findings in Internet addiction. This discrepancy could be explained by an empirical difference in the two entities or could be an epistemological artifact resulting from the *a priori* description of hikikomori as a social disease in Japanese literature. Nevertheless, the fact that hikikomori preceded Internet widespread use by some decade seems to point toward a real difference between the two entities. To the authors’ knowledge, no neuroimagery has ever been done to investigate *hikikomori*.

Hikikomori and Internet addiction have some overlap in their proposed criteria. The two share a lost of interest in school or working and difficulties with interpersonal relationship. A difference between *hikikomori* and Internet addiction irrespective of definition would be the insistence on tolerance and withdrawal symptoms in the latter and the presumption that functional impairment originate from the addiction problem and not the other way around. The two syndromes certainly overlap in some cases, such as the loss of interest for other activities, use of Internet to escape dysphoric mood, and functional impairment (4, 18, 20). Up to 56% of *hikikomori* individuals may be at-risk of Internet addiction and 9% addicted in South Korea (4). For instance, a South Korean study reported that several psychiatrists diagnosed Internet addiction in a case-vignette of a Japanese patient with *hikikomori* (23). In contrast to cases of addiction, the Internet may actually be beneficial for a hikikomori’s quality of life by giving him a way to meet people with common interests and similar problems (42). Such a development could therefore be a sign of improvement and not a complication (or comorbidity). As a result, many treatment facilities use the Internet to manage *hikikomori* because it is often the only acceptable way for them to interact with health professionals (43). In the case of Internet addiction, the criteria suggest that the behaviors are egodystonic and thus leading to suffering, which is not necessarily the case for hikikomori who can view their behavior as part of their identity (egosyntonic).

It is possible in many hikikomori cases to diagnose Internet addiction disorder as comorbid. However, as alluded to previously, many *hikikomori* actually use the Internet adaptively for social interactions (20) as it enables them to identify with others in similar situations and so keep themselves somewhat connected to the outside world (43). From a pragmatic point of view, the question could be what an Internet addiction diagnosis adds to the management of a *hikikomori*. It may be useful if it gives patients access to additional services, but given the scarcity of research on treatment of Internet addiction (44) and novelty of the diagnosis, it would be quite surprising. It would then be prudent not to over-pathologize such behaviors depending on the context, especially with cut-offs still contentious and arbitrary (45).

Thinking the other way around, it seems less likely that a patient presenting for Internet addiction outside of Asia would receive a hikikomori diagnosis because there is an element of self-proclaimed identity in hikikomori that seem to be restricted to this continent. Nevertheless, adding systemic factors thought to be responsible of hikikomori (family conflicts, social transformation, shame in regard to perceived failure, etc.) could benefit some Internet-addicted patients for whom these factors seem to play a major role in their addiction.

Another important diagnosis of exclusion is psychosis, which may be associated with both *hikikomori* (12) and Internet addiction (46). Full-blown schizophrenia is usually preceded by a phase of the prodrome, which may resemble *hikikomori* (47, 48). Symptoms common to both conditions include social isolation, deterioration of functions related to the social role, deterioration of hygiene, loss of drive, anxiety, mistrust, irritability, depressive mood, sleep disorder, and loss of concentration (5, 10, 49). Of particular relevance is the ICD-10 subtype of simple schizophrenia (50), which presents essentially with negative symptoms and odd behaviors without delusions or hallucinations (51), although this diagnosis is controversial and has been dropped from the DSM classification because of poor reliability and lack of use (51).

Two aspects may help in differentiating between the two. First, behavioral oddity is not necessarily present in *hikikomori* and, second, a patient with *hikikomori* may not experience other negative symptoms in addition to social isolation, such as cognitive deficits. As mentioned before, negative symptoms are not specific to psychosis and could suggest other diagnoses such as depression or amotivational syndrome secondary to cannabis use (52).

Sensory deprivation in *hikikomori* who stay for extended periods in their room using the Internet could also lead to a presentation resembling psychosis. Even though in the general population, 13.2–28.4% of people may experience psychotic-like symptoms in their lifetime (53, 54), a recent report showed that in a cohort of 170 university students psychotic-like symptoms over a 2-month period were associated with problematic Internet use (46). The authors argued that Internet use could be a stressor unmasking a vulnerability or, alternatively, that at-risk individuals with interpersonal deficits could spend more time online to meet people (46, 55). This later explanation resembles what has been mentioned earlier about *hikikomori* and Internet use (43). Also, sensory deprivation has been linked to psychotic symptoms for decades even in typical individuals (56). The sensory deprivation resulting from social withdrawal could increase psychotic symptoms in *hikikomori* too, blurring the line between the two diagnosis. In the absence of overt full-blown psychotic symptoms suggestive of an acute episode of psychosis, modifying the environment (reducing sensory deprivation and Internet use, for example) may help in differentiating between *hikikomori*, psychosis, and Internet addiction. The chronological development of symptoms could be another sign of which condition came first and “triggered” the other.

In the clinical experience of one of the authors (Emmanuel Stip), several patients experience at some point a clearly psychotic episode with a theme related to computers or confusion regarding the world of virtual reality games (57). Others have obsessive-compulsive traits. Many also show intense negative symptoms on validated psychiatric scales such as the PANSS with a mean score of 60 on the negative subscale, which are resistant to treatment (57). Eliminating comorbid diagnoses is therefore of paramount importance. However, not all cases are accompanied by another mental disorder or if an illness is observed, the comorbid diagnosis does not sufficiently explain the prolonged withdrawal and social confinement (58).

### Management of *Hikikomori*

Consultation tends to occur late in the course of *hikikomori*, partly because of the nature of the disease – the social withdrawal behavior – and partly because of the family’s resistance to address the issue for reasons of guilt, shame, fear, social stigma, and lack of knowledge. Reaching out with traditional treatment settings may prove difficult and treatment implication of *hikikomori* cases is often one of the major barriers to adequate management (4, 5, 10, 12, 13).

There are three broad types of service providers to help *hikikomori* in Japan: (1) mental health centers that use psychological/clinical approaches; (2) community settings that use non-clinical or psychosocial approaches; and (3) a variety of other settings offering alternative treatment (e.g., horse-assisted therapy, communal cooking in a farm, and online platforms) (19). Services often depend on how *hikikomori* is defined and understood but a comprehensive management plan should include both clinical and social treatments (19). The goal of the management is to break their physical isolation (that is to draw them out of their room or other environment) and social isolation, and then push them to adopt an active role in society, whether that is to return to school or integrate the labor market (5).

In the first instance, management of *hikikomori* entails a comprehensive clinical evaluation to exclude the presence of psychiatric comorbidity. If a comorbidity is present, relevant clinical treatments should be offered. Hospitalization may be necessary in certain cases of serious functional impairment, and appropriate pharmacotherapy and/or psychotherapy for concurrent illnesses, such as schizophrenia, depression, and social phobia, may be indicated. Psychosocial and psychotherapeutic interventions may also be needed for pervasive developmental or personality disorders. Many, however, lack such a psychiatric diagnosis and are considered to be “primary hikikomori.” In these cases, or in cases where comorbid diagnosis are not the major problem or only cause of functional impairment, counseling services, home visitation programs incorporating brief psychotherapy interventions, and family or group therapy show most promise although there are methodological issues with the available evidence (4, 5, 10, 12, 49). Psychodynamic psychotherapy and nidotherapy, the systematic manipulation of the physical and social environment to help achieve a better fit for patients, have also been used (14, 57, 59). Evidence on pharmacotherapy is even scarcer. Paroxetine was successfully used in one patient with obsessive-compulsive disorder who withdrew to his room for 10 years but it is unclear whether this is marks true primary *hikikomori* (10).

Treatment can be lengthy, as complete and sustained engagement in the therapeutic process is uncommon and only a minority of cases achieves full social participation (4, 12, 13, 32).

As a whole, evidence regarding treatment is mostly based on small case series or case report, with a lack of randomized controlled trials (5). It is probably safe to say that clinical treatment should be given if a psychiatric comorbidity is present, but there is no reason that it should be at the exclusion of other types of treatment, as long as they do not interfere with each other. Using an eclectic paradigm with both clinical treatment (with its in-depth knowledge about mental health disease) and psychosocial treatments (with its emphasis on social reintegration, outreach and cultural specificity) could be beneficial to the *hikikomori* with comorbidity (19). Primary *hikikomori* cases would probably benefit most from psychosocial treatment, but a reevaluation by a clinician after some time could ensure that the patient is still not showing sign of psychiatric symptoms.

### Prognosis

Again, this is reflective of the underlying or comorbid disorder. One study showed that patients with both social anxiety disorder and *hikikomori* had a worse prognosis than those with social phobia alone, suggesting that *hikikomori* was an extreme variant of the former.

If a *hikikomori* finally reintegrates voluntarily into ­society – often after several years – he/she faces a serious problem: catching up on the lost years of schooling or work. This makes it more difficult to return to society. The outcomes for individuals with *hikikomori* are much worse if they do not seek help, even if their family members are supportive (13).

## Concluding Remarks

This case seems to fit the description of “*hikikomori* syndrome” or “prolonged social withdrawal syndrome” and we believe that it is the first published report from Canada. The patient did not clearly meet any other psychiatric diagnosis, such as a major depressive episode, an anxiety disorder, or any personality disorder, according to the DSM-5 criteria. It is possible that his symptoms were due to a prodromal phase of psychosis or negative symptoms of schizophrenia although there was little evidence for this diagnosis at presentation or subsequently. Internet addiction was also considered although in this particular case, the intense and prolonged daily use of Internet seemed to have arisen secondarily to his prolonged social withdrawal. In addition, the removal of his computer and Internet access did not cause a change in his behavior or his social withdrawal. Importantly, he was able to resume his work and studies without the need for medication or psychotherapy.

The exact place of *hikikomori* in psychiatric nosology has yet to be determined. One of the questions raised is if this is a separate culture-bound syndrome. Some authors state that it is not a syndrome, but rather an idiom of distress, which could explain the absence of a standard and unanimously accepted clinical description across the scientific literature (58, 60). Some even argue that *hikikomori* might be a non-pathological or dissociative response to distress (42) and be beneficial in terms of social growth and identity construction (6). Emerging behaviors such as *hikikomori* may reflect adolescents’ changing relationship with the environment and the family, especially in view of the consequent social withdrawal and the family’s suffering and powerlessness. While there is controversy as to whether *hikikomori* should be a psychiatric diagnosis or not, *hikikomori* is usually considered a “disorder” by clinicians in Japan (20). However, there is uncertainty over whether *hikikomori* is a primary or secondary disorder (social withdrawal not associated with any underlying psychiatric disorder), or solely a secondary clinical presentation, where social withdrawal is associated with other psychiatric conditions. However, as recently highlighted in the literature (58), adopting a reduced perspective or theoretical frame would probably be a nosological and etiological mistake, especially taking into account the heterogeneous presentation and the limited literature with no clear correlational relationship with any other psychiatric disorder or sociological phenomenon. Clinical practice in programs for initial episodes or in consultation regarding a potential diagnosis of prodromal psychosis leads us to consider various presentations, including those specific to young people of the generation that philosopher Michel Serres nicknamed “Thumbelina”: a new human mutation resulting in the ability to text with their thumbs (61). Schoolchildren and students today are experiencing a tsunami of change and end up spending more time in the virtual than the real world.

Thus, although *hikikomori* can perhaps be presently described as the resulting interaction among psychological, biological, and sociological factors, further research is still needed to distinguish between primary and secondary *hikikomori* and establish whether this is a new diagnostic entity, or particular cultural or societal manifestations of established diagnoses. Cohort studies may assist in establishing environmental or genetic risk factors, whereas randomized controlled trials could improve our understanding of effective treatments. In the meantime, case reports from around the world can help our understanding of this condition and so help operationalize the concept.

## Ethics Statement

Written informed consent was obtained from the subject after complete explanations of the study were provided, including Brain imaging. The study was approved by the Ethics Committee of Fernand Seguin Research Center, in Montréal, QC, Canada. The study presented in the manuscript involved a human subject.

## Author Contributions

ES is first author and the corresponding author. AC, AT, and SK participated in writing section by section and reviewed a first draft.

## Conflict of Interest Statement

The authors declare that the research was conducted in the absence of any commercial or financial relationships that could be construed as a potential conflict of interest.
